# Predictive Factors of Successful Microdissection Testicular Sperm Extraction in Patients with Presumed Sertoli Cell-Only Syndrome 

**DOI:** 10.22074/ijfs.2015.4214

**Published:** 2015-04-21

**Authors:** Tahereh Modarresi, Hani Hosseinifar, Ali Daliri Hampa, Mohammad Chehrazi, Jalil Hosseini, Faramarz Farrahi, Farid Dadkhah, Marjan Sabbaghian, Mohammad Ali Sadighi Gilani

**Affiliations:** 1 Department of Andrology at Reproductive Biomedicine Research Center, Royan Institute for Reproductive Biomedi- cine, ACECR, Tehran, Iran; 2 Department of Epidemiology and Reproductive Health at Reproductive Epidemiology Research Center, Royan Institute for Reproductive Biomedicine, ACECR, Tehran, Iran; 3 Department of Urology, Shariati Hospital, Tehran University of Medical Sciences, Tehran, Iran

**Keywords:** Follicle Stimulating Hormone, Luteinizing Hormone, Sperm Retrieval, Azoo- spermia, Nonobstructive

## Abstract

**Background:**

To evaluate predictive factors of successful microdissection-testicular sperm extraction (MD-TESE) in patients with presumed Sertoli cell-only syndrome (SCOS).

**Materials and Methods:**

In this retrospective analysis, 874 men with non-obstructive azoospermia (NOA), among whom 148 individuals with diagnosis of SCOS in prior biopsy, underwent MD-TESE at Department of Andrology, Royan Institute, Tehran, Iran. The predictive values of follicle stimulating hormone (FSH), luteinizing hormone (LH), and testosterone (T) levels, testicular volume, as well as male age for retrieving testicular sperm by MD-TESE were analyzed by multiple logistic regression analysis.

**Results:**

Testicular sperm were successfully retrieved in 23.6% men with presumed SCOS. Using receiver operating characteristic (ROC) curve analysis, it was shown that sperm retrieval rate in the group of men with FSH values >15.25% was 28.9%. This was higher than the group of men with FSH ≤15.25 (11.8%).

**Conclusion:**

Sperm retrieval rate (SRR) was 23.6% in men with presumed SCOS and FSH level can be a fair predictor for SPR at MD-TESE. MD-TESE appears to be recommendable in such cases (SCOS with high FSH concentration) with reasonable results.

## Introduction

For men with a zero sperm count (azoospermia), testicular biopsy is done to determine if a blockage is present (obstructive azoospermia), or if primary testicular failure [non-obstructive azoospermia (NOA)] is the cause (1,2). Primary testicular failure affects approximately 1% of the population and 10% of those seeking fertility evaluations (3,4). 

The general histological patterns of the patients with non-obstructive azoospermia are hypospermatogenesis, maturation arrest and Sertoli cell– only syndrome (SCOS) (4). 

For many azoospermic men, *in vitro* fertilization/intracytoplasmic sperm injection (IVF/ICSI) has become the major reproductive treatment option if testicular sperm can be retrieved (1). Microdissection testicular sperm extraction (MD-TESE) is an effective sperm retrieval procedure for men with NOA due to higher sperm retrieval rate (5,6). Based on microscopical scale, MD-TESE identifies the most advanced pattern, not necessarily the predominant pattern of spermatogenesis in the testis (5). In contrast to the predominant spermatogenic pattern, the most advanced pattern appears to affect sperm retrieval results. 

The sperm retrieval rates (SRRs) by MD-TESE for patients with hypospermatogenesis were 81 (7) to 100% (8,9) of attempts, whereas in those with maturation arrest spermatozoa were retrieved in only 44 (7) to 75% (8,9) of MD-TESE attempts. In SCOS patients, SRRs were between 22.5 (8) and 41% (7). Since sperm extraction is often scheduled in addition to oocyte retrieval after ovarian stimulation and monitoring, failed ME-TESE can have significant emotional and financial implications for the couples involved. So it would be valuable to predict the success of sperm retrieval using noninvasive parameters (5,10). 

Serum FSH is an indirect reflection of the spermatogenic function of the testis (5,10). Clinically, testicular volume is correlated with spermatogenesis. Age may also affect the outcome of sperm extraction (10). Since one of the most frequent histological patterns characterizing absence of sperm is SCOS, the primary aim of this study was to evaluate the outcomes of ME-TESE, primarily the sperm retrieval, in patients with diagnosis of SCOS in prior biopsy. We also analyzed the predictive values of follicle stimulating hormone (FSH), luteinizing hormone (LH) and testosterone (T) levels, testicular volume, as well as male age for retrieving testicular sperm by MD-TESE in these patients. 

## Materials and Methods

### Patients

In this a retrospective analysis, 874 men with NOA, among whom 148 patients with diagnosis of SCOS in prior biopsy, underwent MD-TESE at Royan Institute, Tehran, Iran, between April 2008 and March 2009. Azoospermia was confirmed by at least two semen analyses according to World Health Organization (WHO) guidelines (11). We excluded patients with karyotype abnormalities and Y chromosome microdeletion. Testicular volume was measured by physical examination using an orchidometer. Analysis of serum FSH, LH, and T levels was done by electrochemiluminescence assay in the morning. The reference ranges for FSH level is 1.0-10.5 mIU/mL, LH level is 1.09.5 mIU/mL and T level is 2.0-12 ng/mL. The men were divided into four main groups based on FSH level in increments of 10 IU/mL as follows: i. <10 mIU/mL, ii. 10-20 mIU/mL, iii. 20-30 mIU/mL, and iv. >30 mIU/mL. In other classification, the patients were classified into two following subgroups based on the best cut point of FSH values (see statistical analysis): i. the group with unsuccessful sperm retrieval and ii. the group with successful sperm retrieval. Approval was obtained from Ethics Committee of Royan Institute for this particular study, and participants provided a written informed consent. 

### Microdissection testicular sperm extraction (MDTESE)

MD-TESE was performed under general anesthesia according to the technique previously described by Schlegel (12). In brief, the scrotum was incised along the scrotal raphe and testis was opened from the mid-part. Using an operating microscope with ×25-40 magnification, enlarged and opaque seminiferous tubules were removed and evaluated by one of our three expert lab technicians in operating room. Each sample was placed in a petri dish filled with 1 mL Ham’s F10 medium (Biochrom, Germany), and was mechanically cut, dispersed and examined under an inverted microscope (Nikon, Japan) at ×400 magnification. If no spermatozoa were seen, microdissection of additional areas of that testicle and contralateral testicle were carried out and subsequent samples were taken. If no sperm was seen in the operating room, all testicular samples were subjected to centrifugation at 3000 rpm with 5 mL Ham’s F10 and examined to determine the presence of even a single sperm. 

### Statistical analysis

Descriptive statistics are presented as mean±SD and percent. Student’s t test (Unpaired) was used to compare mean age, while Mann-Whitney U test was applied to compare testis volume as well as FSH, LH and T levels for outcome of sperm retrieval. Multiple logistic regression analysis was used to evaluate the association between FSH, LH and T levels and success of sperm retrieval, adjusting for potential confounding variables (testicular volume and age). Two tailed p values less than 0.05 were considered statistically significant. We performed receiver operating curve (ROC) analysis for a final model. The area under a curve (AUC) is a measure of predictive power called concordance index which is generated to evaluate the predictive accuracy of selected predictors on probability of retrieving sperm. The value of 0.5 means that predictions are no better than random guessing and the value of 1.0 indicates a (theoretically) perfect test (i.e., 100% sensitive and 100% specific). Moreover, we used ROC analysis to determine the best cut point of FSH level for outcome of sperm retrieval, and sensitivity and specificity were measured 64.3 and 15.4%, respectively. 

## Results

Testicular sperm was successfully retrieved in 23.6% (35/148) of the patients with SCOS. Successful rates for ICSI and clinical pregnancy were 57.1% (20/35) and 9%, respectively. The mean values of age, testicular volume; as well as serum FSH, LH and T levels were compared between those patients with unsuccessful sperm retrieval and the group with successful sperm retrieval. There were no significant differences between two groups (Table 1). 

Findings were shown that there was an inverse linear correlation between FSH and testicular volume (r=-0.37, p<0.001). Sperm retrieval rates in the groups of men with FSH values of 10-20, 2030, and >30 mIU/mL were 39, 37, and 24%, respectively, and this was higher than the group of men with FSH<10 mIU/mL (10%), but this difference was not significant (p>0.05). 

We performed multiple logistic regression analysis with serum FSH, LH and T levels, testis volume and age to predict sperm retrieval during MD-TESE. Adjusted association from the model showed that chance of retrieving sperm during MD-TESE cannot be predicted by any variable. 

**Fig.1:**
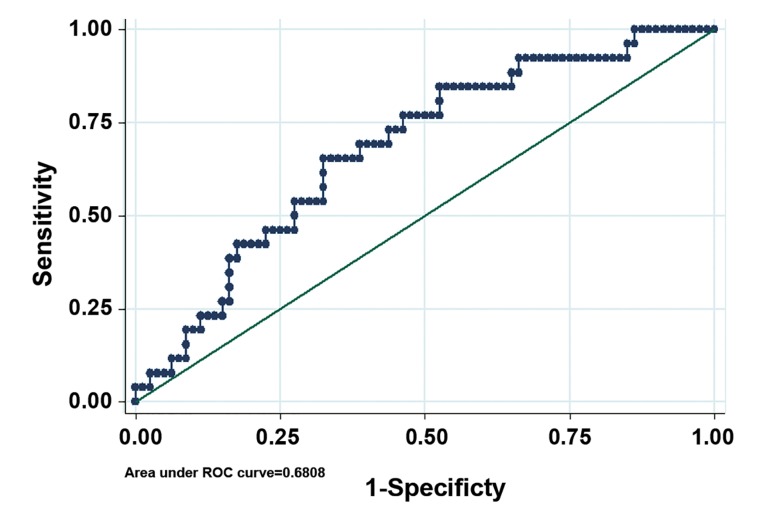
ROC curve of pertinent preoperative parameters to discriminate successful and failed MD-TESE (AUC=0.68). ROC; Receiver operating characteristic, MD-TESE; Microdissection-testicular sperm extraction and AUC; Area under a curve.

Also we used ROC analysis to determine the best cut point of FSH levels for outcome of sperm retrieval. Our results showed that 15.25 was a cut point of FSH for sperm retrieval. SRR in men with FSH>15.25 was 28.9% and in men with FSH≤15.25 was 11.8%. Odds of SRR in men with FSH>15.25 was higher than men with FSH≤15.25 and was significant at level 10%. A logistic regression analysis based on this cut point showed a fair prediction model (AUC=0.68) for FSH (Fig .1). LH level, testosterone level, testicular volume and male age cannot predict presence of sperm with MDTESE (Tables2, 3). 

**Table 1:** Values of age; testicular volume; as well as serum FSH, LH and T levels between two groups (the group with unsuccessful sperm retrieval and the group with successful sperm retrieval)


	Fail		Success		P value
	Mean±SD	CI (%95)	Mean±SD	CI (%95)		

**Age ( Y)**	33±6	31.9-34.2	33±5	31.7-35.6	0.64
**Testicular volume (mL)**	11±4.7	10.1±11.9	9.9±4.3	8.3-11.4	0.24
**FSH (mIU/mL)**	23.7±15.9	20.3-27.2	23.8±9.5	20-27.7	0.36
**LH (mIU/mL)**	9.5±9.1	7.5-11.5	8.6±7.4	5.6-11.6	0.89
**T (ng/mL)**	3.8±2.3	3.3-4.3	4.1±3.5	2.7-5.6	0.84


LH; Luteinizing hormone, T; Testosterone, FSH; Follicle stimulating hormone and CI; Confidence interval.

spermatogenesis. Testicular volume has been found to have poor predictive value for successful TESE (10,13,14). Ramasamy et al. (6) reported that testicular volume didn’t have predictive value to determine the success of MD-TESE, but there was an inverse linear correlation between FSH and testicular volume. Our study confirmed that study and showed that sperm retrieval rate was also higher in smaller testis, but testicular volume didn’t have predictive value to determine the success of MD-TESE. Moreover, in this study, there was an inverse linear correlation between FSH and testicular volume. 

**Table 2:** Baseline characteristics of men with NOA and SCOS


	Serum FSH ( mIU/mL)	
	≤15.25	>15.25

**%**	31%	69%
**Male age (Y)**	32±4	34±6
**Mean FSH (mIU/mL)**	11.2	29.4
**Mean Testosterone (ng/mL)**	4.6±2.6	3.6±2.6
**Mean LH (mIU/mL)**	5.7±6.2	10.2±9.3
**Avg. vol. of testis (mL)**	12.8±4.1	9.6±4.7


LH; Luteinizing hormone, T; Testosterone, SCOS; Sertoli cell-only syndrome, NOA; Non-obstructive azoospermia and FSH; Follicle stimulating hormone.

**Table 3:** Results of multivariable adjusted model of pertinent variables


Variable	P value	OR (95%CI)

**FSH (IU/mL)**		
**≤15.25**		Reference group
**>15.25**	0.067	2.96(0.92-9.4)
**Male age (Y)**	0.648	1.01(0.94-1.1)
**LH (mIU/mL)**	0.224	0.96(0.89-1.02)
**T (ng/mL)**	0.262	1.1 (0.92-1.3)
**Testes size (mL)**	0.103	0.9 (0.8-1.02)


*Adjusted OR represents the estimates from full model adjusted for male age, testes size, LH; Luteinizing hormone, T; Testosterone, FSH; Follicle stimulating hormone levels, CI; Confidence interval and OR; Odds ratio.

## Discussion

MD-TESE has become a recognized procedure for men with NOA. Simultaneous sperm extractiontesticular volume-ICSI exposes the couple to an emotional burden, so it would be beneficial to predict the success of sperm retrieval before treatment. Diagnostic biopsy, hormones levels, volume of testis and age are potentially predictive factors for sperm retrieval (3).

The diagnosis of NOA can only be definitely made on testicular biopsy, but the prognostic value of random biopsy to detect sperm production in these patients is unknown (3). Diagnostic biopsy has limited prognostic value for prediction of sperm retrieval in MD-TESE. Tsujimura et al. (8) reported that SRRs by MD-TESE for SCOS were 22.5%. Gul et al. (13) demonstrated that SRRs for these patients were 27.6%. Okada et al. showed that SRRs by MD-TESE for SCOS were 33.9% (9) and Ramasamy et al. (7) reported excellent SRRs of 41% for SCOS. Our study showed that SRRs by MD-TESE were 23.6% for SCOS.

An important serum parameter in the first years of TESE was the FSH level. In general, the serum concentration of FSH is inversely correlated with impairment of spermatogenesis. Earlier studies showed that elevated FSH levels were associated with a low probability for the retrieval of spermatozoa in TESE (14), but Ramasamy et al. (6) showed that after using MD-TESE, sperm retrieval was higher in NOA men with FSH>15 mIU/mL than those men with FSH<15 mIU/mL. In other study, Ramasamy et al. (15) showed that FSH (and testicular volume) at the repeat MD-TESE appeared to have predictive value to determine the success of a second attempt. Our study confirmed the results of their study. The present study showed sperm retrieval was higher in NOA men with FSH>15.25 mIU/mL than those men with FSH≤15.25 mIU/mL. Also we showed FSH could be a fair predictor of sperm retrieval. Ramasamy et al. (6) have described the reason of the conflicting evidence between TESE and MD-TESE.

We also studied the effect of serum LH and T levels on sperm retrieval, but these factors didn’t have any effect on SRR. This is similar to the results of other studies (13, 16).

Clinically testicular volume is correlated with spermatogenesis. Testicular volume has been found to have poor predictive value for successful TESE (10, 13, 14). Ramasamy et al. (6) reported that testicular volume didn’t have predictive value to determine the success of MD-TESE, but there was an inverse linear correlation between FSH and testicular volume. Our study confirmed that study and showed that sperm retrieval rate was also higher in smaller testis, but testicular volume didn’t have predictive value to determine the success of MD-TESE. Moreover, in this study, there was an inverse linear correlation between FSH and testicular volume.

Many studies have shown a relationship between testicular histopathologic findings and testicular sperm retrieval by TESE (14,17). Histopathologic finding are generally the most useful predictive factor for successful TESE (6). However, it is still controversial whether invasive examination such as testicular biopsy should be performed due to possible presence of inflammatory change, hematoma, fibrosis and devascularization of testes (3,6,9). On the other hand, MD-TESE has fewer postoperative complications than random biopsy (10,16). MD-TESE appears to be recommendable in cases of atrophied testicles and elevated amount of FSH concentration (16). 

The p value for association between FSH (as continuous variable) and sperm retrieval was 0.98. Since FSH is a continuous variable, the odds of sperm retrieval do not change significantly per unit increase in FSH. This conclusion may be clinically expected. So we tried to find a cut point which discriminated sperm retrieval result extremely. ROC analysis was used to find the best cut point for sensitivity and specificity. The association between categorical FSH and sperm retrieval was significant at level of 10%. The p value is near 0.05 in this case. The border p values may be due to sampling errors or small sample size and need further study with larger sample size to find the best cut point. 

## Conclusion

Sperm retrieval rate was 23.6% in men with SCOS and FSH can be a fair predictor of sperm retrieval at MD-TESE. MD-TESE appears to be recommendable in such cases (SCOS with high FSH concentration) with reasonable results. 
